# RT‐CGM in conjunction with CSII vs MDI in optimizing glycaemic control in T1DM: Systemic review and meta‐analysis

**DOI:** 10.1002/edm2.324

**Published:** 2022-02-04

**Authors:** Jimmy William, Jane McCluskey, Nigel Gleeson

**Affiliations:** ^1^ Sligo University Hospital – Haematology Department Sligo Ireland; ^2^ Queen Margaret University Queen Margaret University Way Musselburgh UK

**Keywords:** continuous glucose monitoring, continuous subcutaneous insulin infusions, glycaemic control, multiple daily injections, type 1 diabetes

## Abstract

**Introduction:**

To determine the impact of real‐time continuous glucose monitoring (RT‐CGM) in conjunction with ‘Open loop’‐ continuous subcutaneous insulin infusion (CSII) as compared to conventional multiple daily injections (MDI) in type 1 diabetes.

**Methods:**

We explored the COCHRANE database, MEDLINE, WEB OF SCIENCE, GOOGLE SCHOLARS, PUBMED, EMBASE, and cited literature in articles retrieved (2010–2021) for all randomized controlled trials and real‐world trials of more than 6 months duration in patients with type 1 diabetes that compared RT‐CGM+CSII vs RT‐ CGM+MDI. A total of 1645 publications have been identified; however, only 3 trials fulfilled our inclusion criteria with a total number of 150 patients (72 patients using RT‐CGM+CSII and 78 patients on RT‐CGM+MDI). A Systematic Review and Meta‐analysis were carried out.

**Results:**

No statistically significant reduction in HbA1c was found on comparing RT‐CGM+CSII vs RT‐ CGM + MDI, with *p*‐value = .75. Likewise, impact on TIR, weight and insulin usage was found to be statistically insignificant with *p*‐value of 0.15, 0.75 and 0.20 respectively. There was an overall homogeneity between the 3 trials in respect to all previous variables with *I* ^2^ being 0%.

**Conclusions:**

Real‐time continuous glucose monitors in conjunction with MDI open‐loop CSII had a similar impact on HbA1c, weight, insulin usage and TIR. In addition, RT‐CGM when combined with CSII was associated with higher costs and reduced quality of life, hence RT‐ CGM+MDI can be considered as a cheaper, safer yet equivalent substitute.

**Review Registration:**

This study was registered in PROSPERO (International prospective register of systematic reviews). Registration Name: RT‐CGM in conjunction with CSII vs MDI in optimizing glycaemic control in T1DM: a systematic review. Registration No: CRD42021255333. Accessible at: https://www.crd.york.ac.uk/prospero/display_record.php?ID=CRD42021255333. Amendments: Few amendments to the above‐mentioned registration were made: (1) Title (Meta‐analysis was added). (2) Prof. Gleeson was added as an author. (3) Real‐world trials were included. (4) Outcomes required in studies as per our inclusion criteria amended to include at least 1 outcome. (5) Bias risk was assessed by the CASP tool.

## Graphical abstract

This is the first systematic review and meta‐analysis comparing RT‐CGM+CSII vs RT‐CGM+MDI and its impact on glycaemic control. It is a recap of the last 22 years' novel interventions in the field of diabetes. The chief result of our meta‐analysis is that RT‐CGM in conjunction with CSII or MDI has a similar impact on the glycaemic outcome, weight, insulin usage and TIR.

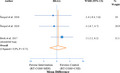

## 1 INTRODUCTION

In 2019, the global estimate of people with diabetes was 463 million. This number is projected to reach 578 million by 2030 and 700 million by 2045.^1^ Of these, approximately 10% have type 1 diabetes.^1^ Historically, the first commercially available self‐monitoring blood glucose (SMBG) device became available early in the 1980s, and later in 1999, the FDA approved the first CGM.^2^ Nonetheless, it was not until 12 years later that the Endocrine Society had released its first CGM guidelines in the year 2011 and recommended its use due to data showing its effectiveness in adults with type 1 diabetes, who can utilize these devices on a nearly daily basis.^3^

Recently, the Advanced Technologies & Treatments for Diabetes (ATTD) consensus established recommendations for the relevant aspects of CGM data utilization and reporting among the various diabetes populations and endorsed a standardized template (Ambulatory Glucose Profile) for data presentation and visualization to help unify the information in question.^4^ Furthermore, lately, Continuous Subcutaneous Insulin Infusions (CSII) and Sensor‐Augmented Insulin‐Pump (SAP) with a threshold‐suspend feature have been introduced, allowing automated suspension of basal insulin delivery in response to a predicted or detected low glucose level, which has been found to exhibit eminent results in terms of glycaemic control.^5^

Nevertheless, with the implementations of CGM in conjunction with intensive insulin therapy Sensor‐Augmented Insulin Regimen (SAIR) using CSII or MDI, a new uncertainty was recognized; which method of SAIR has better outcomes, is more cost‐effective and has less frequent hypoglycaemic episodes?

A non‐randomized prospective real‐life clinical trial by Rodbard et al.^6^ compared glycaemic outcome when CGM in used with CSII vs MDI and concluded that both therapy groups have similar and statistically indistinguishable responses with an improvement of glycaemic variability mean by 15.02% and 11.39% respectively.

Furthermore, a multicentre, open‐label, randomized controlled trial involving 15 paediatric National Health Service (NHS) diabetes services in England and Wales, with participants aged between 7 months and 15 years comparing CSII to MDI, concluded that during the first year following type 1 diabetes diagnosis, no clinical benefit of CSII over MDI was identified in children and young people in the UK setting, and treatment with either regimen was suboptimal in achieving HbA1c thresholds. In addition, CSII was not cost‐effective with a mean total cost being higher by £1863 (95% confidence interval £1620 to £2137) for CSII than for MDI; with the most of this difference (£1177) from the additional cost of consumables and devices (undiscounted annual cost of £600 for CSII vs £80 for MDI).^7^ This was contrary to both, STAR 3 study and the Eurythmic Trials, which concluded that switching from optimized MDI to SAP therapy allowed rapid and safe A1c reduction that was observed by 3 months and persisted throughout the study period (18 months).^8^, ^9^ Nevertheless, in both trials, only the patients using CSII were on CGM, and therefore, conclusions were not specific whether glycaemic improvement was due to CGM or CSII use.

The up‐to‐date recommendation from the ADA in 2021^10^ concluded that RT‐CGM or intermittently scanned CGM (isCGM) in conjunction with MDI or CSII can lower and maintain HbA1c, and reduce hypoglycaemia in youth and adults with diabetes to replace SMBG when used properly. However, no consensus is yet available to guide the selection of the appropriate insulin administration method and it continues to be individually based, although socioeconomic status, in addition to other factors like race, ethnicity, private health insurance and education influence this decision.^11^

Therefore, conducting a Systematic Review/Meta‐analysis on the most recent high‐quality studies will aid us in answering our queries concerning the impact of RT‐CGM when combined with CSII as compared to MDI on glycaemic control, cost‐effectiveness, and rates of hypoglycaemia. This will enhance our practice in the field of diabetes management, the quality of life of the diabetic population worldwide and achieve required glycaemic targets by utilizing the most recent innovations.

## 2 AIM & OBJECTIVES

Our primary objective is to assess the impact of RT‐CGM+open‐loop CSII vs RT‐CGM+MDI on HbA1c/GV in type 1 diabetes. However, our secondary objectives are to assess the impact on several other variables:

1. Weight/ BMI
2. Insulin usage
3. TIR, TAR, TBR
4. Severe Hypoglycaemic episodes or ketoacidosis episodes
5. Quality‐adjusted life years
6. The burden of diabetes education employed
7. Cost‐effectiveness

## 3 METHODOLOGY

### 3.1 Study design and setting

This study is a systemic review and meta‐analysis comparing RT‐CGM in combination with CSII vs MDI in type 1 diabetes patients. It includes RCTs and real‐world trials comparing variables in question as per our objectives.

### 3.2 Search strategy

A comprehensive review of the existing literature was undertaken using the Preferred Reporting Items for Systematic Reviews and Meta‐analysis—PRISMA guidelines.^12^ Several search engines were explored using specific keywords as shown below to obtain required RCTs/ real‐world clinical trials. For each database, distinct and comprehensive search strategies were constructed using subject‐heading mapping. In addition, a repeat search was made just before the final analyses and adaptations for British and American English were made for all searches.

### 3.3 Bibliographic databases

1. Queen Margaret online Library
2. Cochrane Central Register of Controlled Trials (Central)
3. MEDLINE
4. Web of Science
5. Google scholars
6. PubMed
7. EMBASE
8. References lists of included trials were checked to identify any additional studies

### 3.4 List of keywords

1. For Glucose Monitoring (CGM* OR Continuous Glucose Monitoring OR RT‐ CGM* OR Real‐time‐ Continuous Glucose Monitoring)
2. For Insulin Delivery (CSII* OR Continuous Subcutaneous Insulin Infusion OR MDI* OR Multiple daily injections)
3. For Type of study (RCT* OR Randomized Controlled Trial OR Systemic review* OR Real‐world clinical trial*)
4. For Type of Diabetes (T1DM* OR Type 1 Diabetes and NOT T2DM* OR Type 2 Diabetes).

### 3.5 Study selection

Publications were eligible if they fulfilled the inclusion criteria, in addition to reporting at least one of the outcomes in question.

### 3.6 Inclusion criteria

1. Prospective RCTs or Real‐world clinical trials published from the year 2010–2021
2. Full text available
3. Written in the English language
4. Clear Inclusion and exclusion criteria
5. Study period ≥6 months
6. RT‐CGM use
7. The age of participants presented
8. Compliance of participants during the trial
9. Weight/BMI pre‐and post‐intervention
10. Insulin usage clearly quantified

Outcomes of interest are pre‐ and post‐intervention values of HbA1c/GV, severe hypoglycaemic episodes and ketoacidosis episodes/ TIR, in addition, cost‐effectiveness, quality‐adjusted life years and burden of diabetes education employed.

### 3.7 Exclusion criteria

1. RCTs/ Real‐world clinical trials not fulfilling the entire inclusion criteria.
2. RCTs/ Real‐world clinical trial including patients with type 2 diabetes.

### 3.8 Data extraction

Through the search strategy being implemented only articles that fulfilled the inclusion criteria were included in the study. Included trials were downloaded, and data were extracted from each study using customized data extraction forms created to include relevant data required for subsequent data analysis (Tables 1, 2, 3).

**TABLE 1:** Customized data extraction form of Šoupal et al. 2016

Study	Comparison of different treatment modalities for type 1 diabetes, including sensor‐augmented insulin regimens, in 52 weeks of follow‐up: a COMISAIR study. (Jan Šoupal, et al., 2016)			
	CGM/CSII		CGM/MDI	
Sample size (Mean/SD)	15		12	
Study period	52 weeks		52 weeks	
Randomization/Blinding	Non‐randomized prospective		Non‐randomized prospective	
Mean age (Mean/SD)	33 ± 10		34 ± 10	
Gender (Mean)	M: 9 (60%)	F: 6 (40%)	M: 7 (58%)	F: 5 (42%)
Type of insulin	N/A		N/A	
Cost‐effectiveness	N/A		N/A	
QALY	N/A		N/A	
Burden of diabetes education employed	N/A		N/A	

	Wt/kg	BMI	HbA1c % (mmol/mol)	GV	TIR%	TAB%	TBR%	Severe hypoglycaemic episodes	Ketoacidosis episodes	Insulin utilized/day (mean/SD)
Baseline CGM/CSII (Mean/SD)	76.1 ± 10	25 ± 3	8.2 ± 0.9 (66 ± 9)	_	_	_	_	_	_	45 ± 12
Baseline CGM/MDI (Mean/SD)	79.6 ± 13	25 ± 3	8.5 ± 1.1 (69.3 ± 8)	_	_	_	_	_	_	48 ± 12
Post CGM/CSII (Mean/SD)	77.3 ± 9	_	7.1 ± 0.9 (53.9 ± 10)	_	_	_	_	0	0	47 ± 13
Post CGM/MDI (Mean/SD)	81 ± 14	_	7.2 ± 0.8 (55.3 ± 8.7)	_	_	_	_	0	0	50 ± 13

**TABLE 2:** Customized data extraction form of Šoupal et al. 2020

Study	Glycaemic outcomes in adults with T1D are impacted more by continuous glucose monitoring than by insulin delivery method:3 years of follow‐up from the COMISAIR study. (Jan Šoupal, et al., 2020)			
	CGM/CSII		CGM/MDI	
Sample size (Mean/SD)	26		22	
Study period	3 years		3 years	
Randomization/Blinding	Non‐randomized, prospective		Non‐randomized, prospective	
Mean age (Mean/SD)	32.3 ± 9.9		32.6 ± 11.5	
Gender (Mean)	M: 13 (50%)	F:13 (50%)	M: 13 (59%)	F:9 (41%)
Type of insulin	N/A		N/A	
Cost‐effectiveness	N/A		N/A	
QALY	N/A		N/A	
Burden of diabetes education employed	N/A		N/A	

	Wt/kg	BMI	HbA1c % (mmol/mol)	GV SD*T* (mmol/L)	TIR%	TAB%	TBR%	Severe hypoglycaemic episodes	Ketoacidosis episodes	Insulin utilized/day (Mean/SD)
Baseline CGM/CSII (Mean/SD)	72.5 ± 15	25 ± 4	8.2 ± 0.9 (66.5 ± 10.2)	4 ± 0.8	50.9 ± 11.8	_	_	_	_	46.2 ± 11.5
Baseline CGM/MDI (Mean/SD)	76.6 ± 14	26 ± 4	8.2 ± 0.9 (66.6 ± 10.0)	4 ± 0.7	48.7 ± 9.8	_	_	_	_	48.1 ± 15
Post CGM/CSII (Mean/SD)	75.9 ± 10		6.9% (52)	3 ± 0.6	72.3 ± 9.7	_	_	1	1	46.3 ± 10
Post CGM/MDI (Mean/SD)	78.3 ± 16		7.0% (53)	3 ± 0.5	69 ± 12	_	_	1	0	49.7 ± 11

**TABLE 3:** Customized data extraction form of Beck et al. 2017

Study	Effect of initiating use of an insulin pump in adults with type 1 diabetes using multiple daily insulin injections and continuous glucose monitoring (DIAMOND): a multicentre, randomized controlled trial. (Beck et al. 2017)			
	CGM/CSII		CGM/MDI	
Sample size (Mean/SD)	37		38	
Study period	28 weeks		28 weeks	
Randomization/Blinding	RCT non‐blinded		RCT non‐blinded	
Mean age (Mean/SD)	46 ± 15		45 ± 12	
Gender (Mean)	M: 21 (47%)	F:16 (43%)	M: 19 (50%)	F:19 (50%)
Type of insulin (n)	Lispro (19), Aspart (18) Afrezza (1), glargine (29), detemir (4), Isophane (NPH) insulin (1)		Lispro (23), Aspart (15) Afrezza (1), glargine (27), detemir (7), degludec (1), Isophane (NPH) insulin y(1)	
Cost‐effectiveness	Not cost‐effective (Wan et al. 2019)			
QALY	Decrease QALYs by 0.71 (Wan et al. 2019)			
Burden of diabetes education employed	N/A		N/A	

	Wt/kg	BMI	HbA1c % (mmol/mol)	GV%	TIR% (Min/da y)	TAB%	TBR%	Severe hypoglycaemic episodes	Ketoacidosis episodes	Insulin utilized/day (mean/SD)
Baseline CGM/CSII (Mean/SD)	87 ± 14	29 ± 3	7.6 ± 0.7 (60 ± 7.7)	49%	(49%) 708 ± 162	_	_	_	_	62.9 ± 22.6
Baseline CGM/MDI (Mean/SD)	83 ± 18	27 ± 5	7.6 ± 0.9 (60 ± 9.8)	53%	(53%) 762 ± 224	_	_	_	_	54.9 ± 19.6
Post CGM/CSII (Mean/SD)	86 ± 14		7.9 ± 0.7 (63 ± 7.7)	55%	(55%) 795 ± 182	_	_	0	1	_
Post CGM/MDI (Mean/SD)	83 ± 18		7.7 ± 1.0 (61 ± 10.9)	51%	(52%) 746 ± 223			1	0	_

### 3.9 Assessment of risk of bias

CASP tool (accessed 1st September 2021), was utilized to help critically appraise articles and reduce the risk of bias (Table 4). The CASP tool contains checklists that assist researchers in critically appraising various types of evidence. All studies that were included in this review have been deemed credible and non‐prejudiced through CASP tool's checklists.

**TABLE 4:** CASP checklist for qualitative research—Qualitative appraisal tool.

CASP COHORT	Šoupal et al. 2016	Šoupal et al. 2020	CASP RCT	Beck et al. 2017
Did the study address a clearly focused issue?	±	+	Did the trial address a clearly focused issue?	+
Was the cohort recruited in an acceptable way?	+	+	Was the assignment of patients to treatments Randomized?	±
Was the exposure accurately measured to minimize bias?	±	±	Were all of the patients who entered the trial properly accounted for at its conclusion?	+
Was the outcome accurately measured to minimize bias?	±	±	Were patients, health workers and study personnel ‘blind’ to treatment?	−
Have the authors identified all‐important well‐confounding factors?	±	±	Were the groups similar at the start of the trial?	+
Have they taken account of the well‐confounding factors in the design and/or analysis?	±	±	Aside from the experimental intervention, were the groups treated equally?	+
Was the follow‐up of subjects complete enough?	+	+	How large was the treatment effect?	+
Was the follow‐up of subjects long enough?	+	+	How precise was the estimate of the treatment effect?	+
What are the results of this study?	+	+	Can the results be applied in your context?	−
How precise are the results?	±	±	Were all clinically important outcomes considered?	±
Do you believe the results?	±	+	Are the benefits worth the harms and costs?	±
Can the results be applied to the local population?	+	±		
Do the results of this study fit with other available evidence?	+	±		
What are the implications of this study for practice?	+	+		
Total	10.5	10.5		7.5

Abbreviations: “+” meaning yes; “±” meaning partially present; “−” meaning no/can't tell.

### 3.10 Data synthesis

Data were processed through meta‐analysis and represented in a forest plot, with a primary outcome of interest being HbA1c/ GV. Furthermore, the treatment effect was estimated with a mean difference in the ultimate values of HbA1c/GV between the RT‐CGM+CSII group and the RT‐CGM+MDI group.

*I* ^2^ statistics were measured to assess heterogeneity across the studies. MIX 2.0 Pro (version 2.014) was used to yield the data, and 95% confidence intervals (95% CI) was calculated. The mean difference (MD) as an indicator of efficacy has been reported.

## 4 RESULTS

### 4.1 Study selection

**FIGURE 1:**
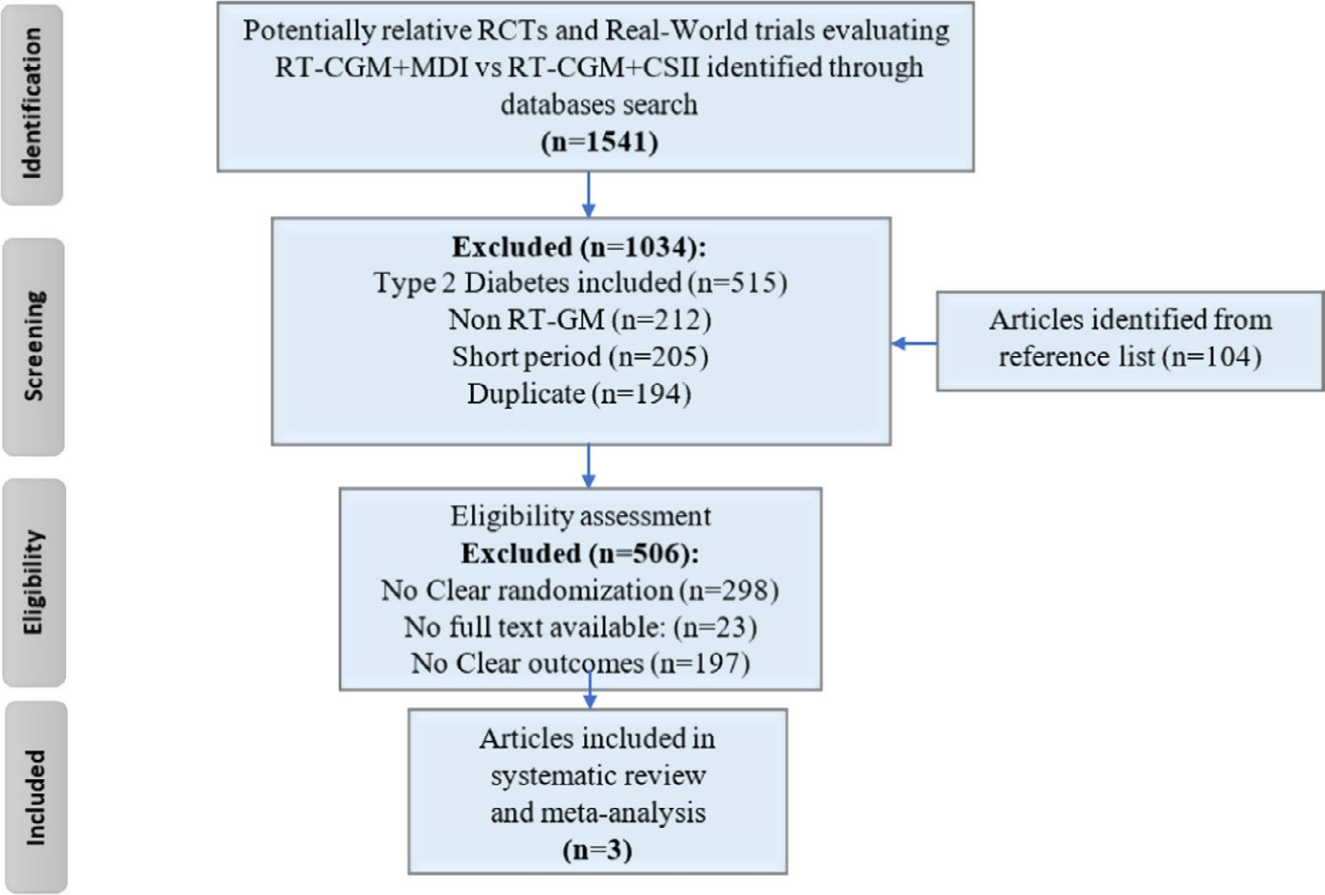
Systematic review and meta‐analysis flow chart

The literature search based on the specific keywords yielded 1541 references, with additional 104 articles identified from reference lists. However, after further assessment of articles as per inclusion criteria, only 3 studies^13^, ^14^, ^15^ were found eligible. Figure 1 schematically demonstrates the search strategy used to identify trials for inclusion.

### 4.2 Quality appraisal and publication bias

All 3 included studies have shown high quality through CASP tool, showing a good methodological approach. Nevertheless, as it is not possible to blind the participants or the experimenters from intervention protocol, all studies did not score in this criterion.

Moreover, the likelihood of publication bias was not feasible to assess since only a small number of studies were eligible to be included in our meta‐analysis.

### 4.3 Participant characteristics

The overall sample size was 150, distributed into two groups, RT‐CGM+CSII and RT‐CGM+MDI (78 and 72) respectively. The mean age of the participants in the included trials was 37.1 years (range 32.3–46 years). In addition, the mean in HbA1c in overall RT‐CGM+CSII and RT‐CGM+MDI groups was 63.3 ± 9.2 (mmol/mol) and 63.5 ± 10.2 (mmol/mol) respectively. More emphasis on the overall characteristics of trials included in the meta‐analysis is provided in Table 5.

**TABLE 5:** Characteristics of trials included in the meta‐analysis

First author, year	Study design	Duration	Sample CGM+CSII/CGM+MDI	Mean age ± SD CGM+CSII/CGM+MDI	Baseline HbA1c% (mmol/mol) CGM+CSII/CGM+MDI	Baseline weight in kg CGM+CSII/CGM+MDI	Primary outcome
Šoupal, 2016	Non randomized, prospective	52 weeks	12/15	33 ± 10/34 ± 10	8.2 ± 0.9 (66 ± 9)/8.5 ± 1.1 (69.3 ± 8)	76.1 ± 10/79.6 ± 13	Efficacy of RT‐CGM with either CSII or MDIs on glycaemic control
Šoupal, 2020	Non randomized, prospective	3 years	22/26	32.3 ± 9.9/32.6 ± 11.5	8.2 ± 0.9 (66.5 ± 10.2)/8.2 ± 0.9(66.6 ± 10.0)	72.5 ± 15/76.6 ± 14	Difference in A1C between the groups after 3 years of follow‐up
Beck, 2017	Open label, RCTs	28 weeks	38/37	46 ± 15/45 ± 12	7.6 ± 0.7 (60 ± 7.7)/7.6 ± 0.9 (60 ± 9.8)	87 ± 14/83 ± 18	Change in CGM‐measured TIR from baseline after the first 4 weeks of the trial

### 4.4 Synthesis of results

**FIGURE 2:**
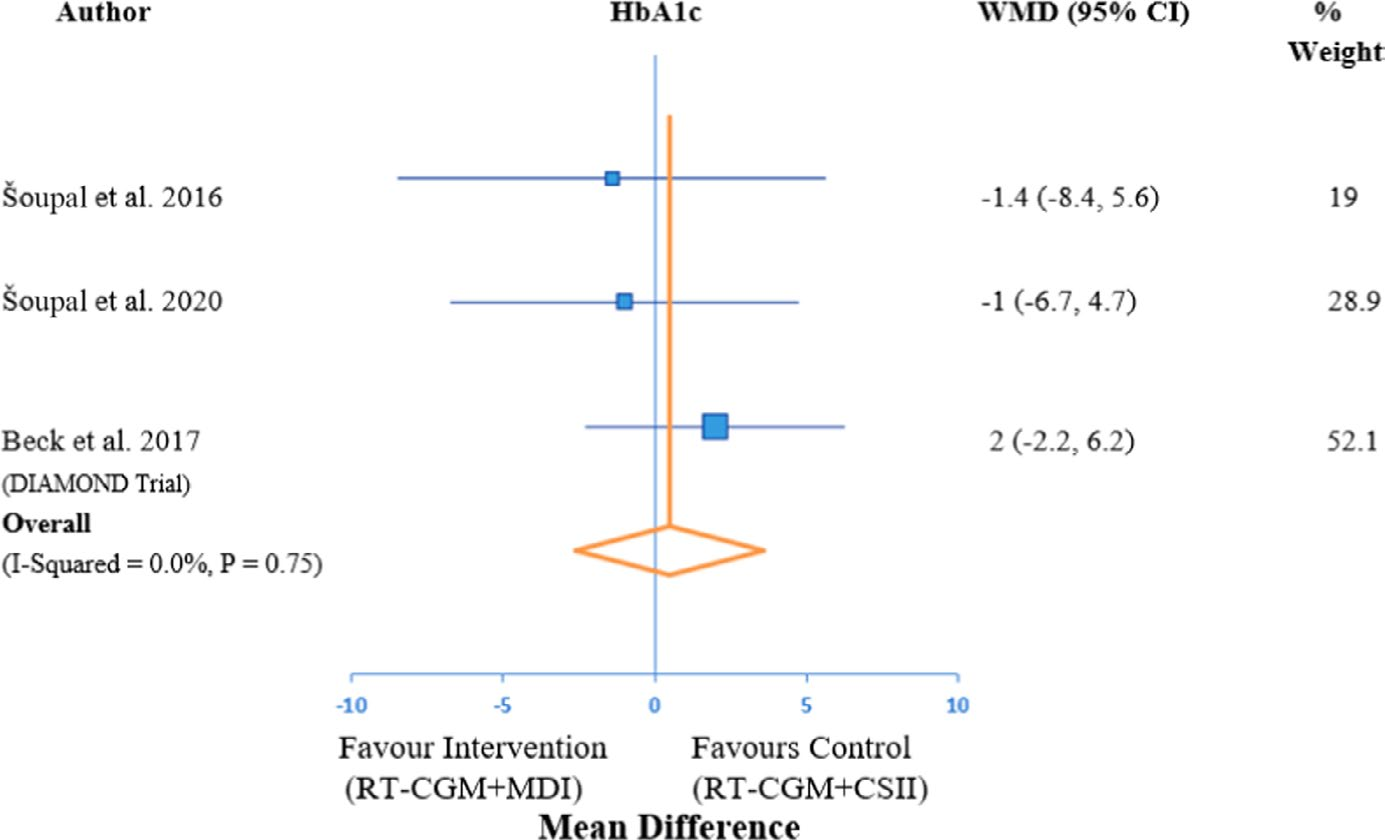
Forest Plot of impact effect of RT‐CGM + CSII vs RT‐CGM + MDI on HbA1c. Effects are shown with a 95% confidence interval (95% CI). WMD, weighted mean difference

Our primary outcome was to assess the impact difference on HbA1c in Type 1 diabetes between patients on RT‐CGM+MDI compared with those on RT‐CGM+CSII through carrying out a meta‐analysis on the data extracted from studies in question. Upon assessment of the results from the 3 trials included, there was no statistically significant reduction in HbA1c on comparing RT‐CGM+CSII vs RT‐CGM+MDI with homogeneity in outcome throughout all studies (*I* ^2^ = 0%, *p*‐value =.75) as shown in Figure 2.

Furthermore, regarding our secondary outcomes, the impact of RT‐CGM+CSII in comparison to RT‐CGM+MDI on TIR, weight and insulin usage, were the only assessable outcomes in our meta‐analysis due to the limited data availability in the included studies. The impact on all these variables was found to be statistically insignificant with p‐value being 0.15, 0.75 and 0.20 respectively (Fig. S1–S3). In addition, all three studies have shown homogeneity when compared to each other in respect to all previous variables with *I* ^2^ being 0% (0%–40% generally considered unimportant; 50%–90%, substantial; Fig. S4).

Nevertheless, since the results within the meta‐analysis have shown overall homogeneity and considering the small number of studies and the relatively low combined sample size, further covariate analysis to reduce in‐group error and eliminate confounding factors will probably be affected and therefore less useful.

## 5 DISCUSSION

This systematic review located 3 trials comparing the use of RT‐CGM+CSII vs RT‐ CGM+MDI. Our main emphasis was to assess the impact on glycaemic control, evaluated by change in HbA1c from baseline, in addition to other secondary outcomes. Although, the extracted sample size of our study is small (*n* = 150), indicating that it may be insufficient to prevent the occurrence of a type 2 error; however, the overall methodological quality of the included studies was ‘good’.

In our study, the overall change in HbA1c from baseline was found to be statistically insignificant upon comparing RT‐CGM+CSII and RT‐CGM+MDI, with the *p*‐value being .75 with inclusive homogeneity. Similar results were observed by Garg et al.,^16^ which is a prospective, non‐randomized prospective real‐life clinical trial that compared CGM in combination with CSII vs MDI and concluded that both therapy groups have similar changes in mean glucose and glucose variability indexes at 3 and 6 months (Intention‐to‐treat analysis, *p* > .05). Nevertheless, Beck et al.^15^ does report that the larger reduction in HbA1c appears to be related to the CGM element rather than with a pump relative to those using MDI and SMBG.

Another variable assessed in our meta‐analysis was the impact on weight, which was found to be statistically insignificant with *p*‐value =.75 and overall heterogeneity of *I* ^2^ = 0%. Impact on weight was not previously assessed in other similar trials utilizing RT‐CGM, however, similar results were obtained by Misso et al.,^17^ which is a systematic review and meta‐analysis comparing the effects of CSII compared to MDI in people with type 1 diabetes mellitus using SMBG.

Moreover, on further assessment of the impact of RT‐CGM+CSII and RT‐CGM+MDI on TIR, results have shown no significant effect with *p*‐value =.1 and *I* ^2^ being 0%. Although, ostensibly contrary to a study by Cherubini et al.,^18^ which concluded that simultaneous use of RT‐CGM+CSII was associated with a higher percentage of TIR, lower time above range >180 mg/dl and lower HbA1c, it was rather a cross‐sectional study on children <18 years old with type 1 diabetes and it did not assess TIR change from baseline but compared results to other insulin delivery and CGM modalities (isCGM+MDI, RT‐CGM+MDI, isCGM+CSII, and RT‐CGM+CSII). Additionally, similar results have been reached upon assessment of impact on insulin usage, were *p*‐value was found to be .20 and *I* ^2^ being 0%, thus there was no significant change in insulin usage between RT‐CGM+CSII or RT‐CGM+MDI.

Nonetheless, Wan et al.,^19^ which was a cost‐effective study of the DIAMOND trial,^15^ showed that within‐trial cost‐effectiveness analysis (CEA) when adding CSII to CGM users increased costs with 28‐week costs were $8272 (RT‐CGM+CSII) vs $5623 (RT‐ CGM+MDI), with the difference in costs being attributed to pump use ($2644). In addition, it reduced quality of life, worsened glucose control (higher HbA1c), caused overall clinical harm and increased non‐severe hypoglycaemic events (NSHEs), which was defined as the detection of a glucose value <3.0 mmol/l (<54 mg/dl) for at least 20 consecutive minutes. As there was only one cost‐effectiveness and QALY study, comparison with other studies was not feasible. However, this was contrary to another study, which concluded that there may be a benefit in using CSII over MI for improving glycaemic control and improving health‐related quality of life in people with type 1 diabetes and non‐severe hypoglycaemic events do not appear to be influenced differently by either intervention, nevertheless, the overall quality of studies in this study was poor, therefore, such variation would be expected.^17^

Another secondary outcome in question was to explore whether RT‐CGM+CSII and RT‐CGM+MDI would increase the frequency of severe hypoglycaemic episodes or ketoacidosis episodes; however, because these unfavourable complications were uncommon in all 3 trials, it was not explored.

### 5.1 Limitations

This current study adds to the existing literature of the most comprehensive accumulation of published evidence regarding RT‐CGM when combined with CSII or MDI and their impact on major outcomes in type 1 diabetes patients. The strength of this meta‐analysis is that it followed the PRISMA guidelines and included only high‐quality studies. Several factors must be considered with respect to the conclusions that can be extrapolated from this study.

Although a wide‐ranging literature search for eligible studies was conducted, other studies may exist. Study selection was based on predetermined inclusion criteria, and only one author assessed full‐text articles for eligibility, thus potentially introducing bias in study selection; however, with our inclusive inclusion criteria and the rather small number of trials highlighting our topic, this may have been alleviated. Our main limitation was the scarcity of the studies emphasizing on comparing RT‐CGM+CSII vs RT‐CGM+MDI. Moreover, only open‐loop insulin delivery with CGM for CSII was reviewed here, although the newer closed‐loop insulin pumps, which are rapidly becoming the standard for CSII are likely to have improved glycaemic outcomes vs RT‐CGM+MDI; however, closed‐loop insulin pump therapy has not been compared in a clinical trial to RT‐CGM+MDI, and thus, no such trials could have been included in this meta‐analysis. In addition to the fact that not all substantial variables were thoroughly explored by included studies, thus influencing the ability to fully explore our study and hindered assessment of a portion of allocated secondary outcomes like the impact on TAR, TBR and glycaemic variance, which are the novel metrics in the field of diabetes and prohibiting more robust exploration of the burden of diabetes education employed impact on the glycaemic outcome.

## 6 CONCLUSION

To the best of our knowledge, this is the first systematic review and meta‐analysis comparing RT‐CGM+CSII vs RT‐CGM+MDI and its impact on glycaemic control, in addition to other significant outcomes. It is a recap of the last 22 years' novel interventions in the field of diabetes, since continuous glucose monitors FDA approval, looking at the impact of RT‐CGM when combined with CSII as opposed to MDI in optimizing glycaemic control in type 1 diabetes. The chief result of our meta‐analysis is that RT‐CGM in conjunction with open‐loop CSII or MDI has a similar impact on the glycaemic outcome, weight, insulin usage and TIR. In addition, RT‐CGM when combined with open‐loop CSII is not cost‐effective with RT‐CGM+MDI being an equally effective alternative. We believe that our results will be of significant relevance in directing future guidelines and recommendations addressing the utilization of SAIR. Nevertheless, further high‐quality RCTs are required to augment our conclusions and explore the benefits and superiority of different sensor‐augmented insulin regimens, particularly those using novel closed‐loop insulin pump technology with glucose control algorithms, to enhance current guidelines and improve the overall disease outcomes.

## CONFLICT OF INTEREST

No conflict of interest that could be perceived as prejudicing the impartiality of the research reported.

## AUTHOR CONTRIBUTION

**Jimmy William:** Conceptualization (lead); Data curation (lead); Investigation (lead); Methodology (equal); Project administration (equal); Resources (equal); Validation (equal); Writing – original draft (lead); Writing – review and editing (equal). **Jane McCluskey:** Conceptualization (equal); Project administration (equal); Resources (equal); Supervision (equal); Validation (equal); Visualization (equal); Writing – review and editing (equal). **Nigel Gleeson:** Formal analysis (lead); Methodology (equal); Resources (equal); Software (equal).

## Supporting information

Fig S1‐S4

## Data Availability

The authors declare that all the data supporting the findings of this study are available within the article and its Supplementary Information files.
