# Supplementary material for: RT‐CGM in conjunction with CSII vs MDI in optimizing glycaemic control in T1DM: Systemic review and meta‐analysis
Source: Endocrinol Diabetes Metab. 2022 Feb 4;5(2):e00324. doi: 10.1002/edm2.324 (PMC8917862; doi:10.1002/edm2.324)
Supplement: Supplementary file 1 — Fig S1‐S4 [file EDM2-5-e00324-s001.docx]

# Supplementary Documents:

**Author Weight WMD (95% CI) % Weight**

Šoupal et al. 2016 -1.4 (-8.4, 5.6) 19%

**-10**

**-5**

**0**

**md**

**5**

**10**

Šoupal et al. 2020 -1 (-6.7, 4.7) 28.8%

Beck et al. 2017 (DIAMOND Trial)

**Overall**

(I-Squared = 0.0%, P = 0.75)

Favour Intervention Favours Control

(RT-CGM+MDI) (RT-CGM+CSII)
 **Mean Difference**

2 (-2.2, 6.2) 52.1%

**Fig S1:** Forest Plot of impact effect of RT-CGM+CSII vs RT-CGM+MDI on Weight.

Effects are shown with 95% confidence interval (95% CI).

WMD: Weighted mean difference.

**Author Inulin Usage WMD (95% CI) % Weight**

Šoupal et al. 2016 -3 (-12.8, 6.8) 26.9%

**-20**

**-10**

**0**

**10**

**md**

Šoupal et al. 2020 -3.4 (-9.3, 2.5) 73.1%

**Overall**

(I-Squared = 0.0%, P = 0.20)

Favour Intervention Favours Control

(RT-CGM+MDI) (RT-CGM+CSII)
 **Mean Difference**

**Fig S2:** Forest Plot of impact effect of RT-CGM+CSII vs RT-CGM+MDI on Insulin Usage. Effects are shown with 95% confidence interval (95% CI).

WMD: Weighted mean difference.

**Author Weight WMD (95% CI) % Weight**

Šoupal et al. 2020 3.1 (-3.1, 9.3) 51.1%

**-5**

**0**

**5**

**md**

**10**

**15**

Beck et al. 2017 (DIAMOND Trial)

**Overall**

(I-Squared = 0.0%, P = 0.15)

Favour Intervention Favours Control

(RT-CGM+MDI) (RT-CGM+CSII)
 **Mean Difference**

3.4 (-2.9, 9.7) 48.9%

**Fig S3:** Forest Plot of impact effect of RT-CGM+CSII vs RT-CGM+MDI on TIR. Effects are shown with 95% confidence interval (95% CI)

WMD: Weighted mean difference.


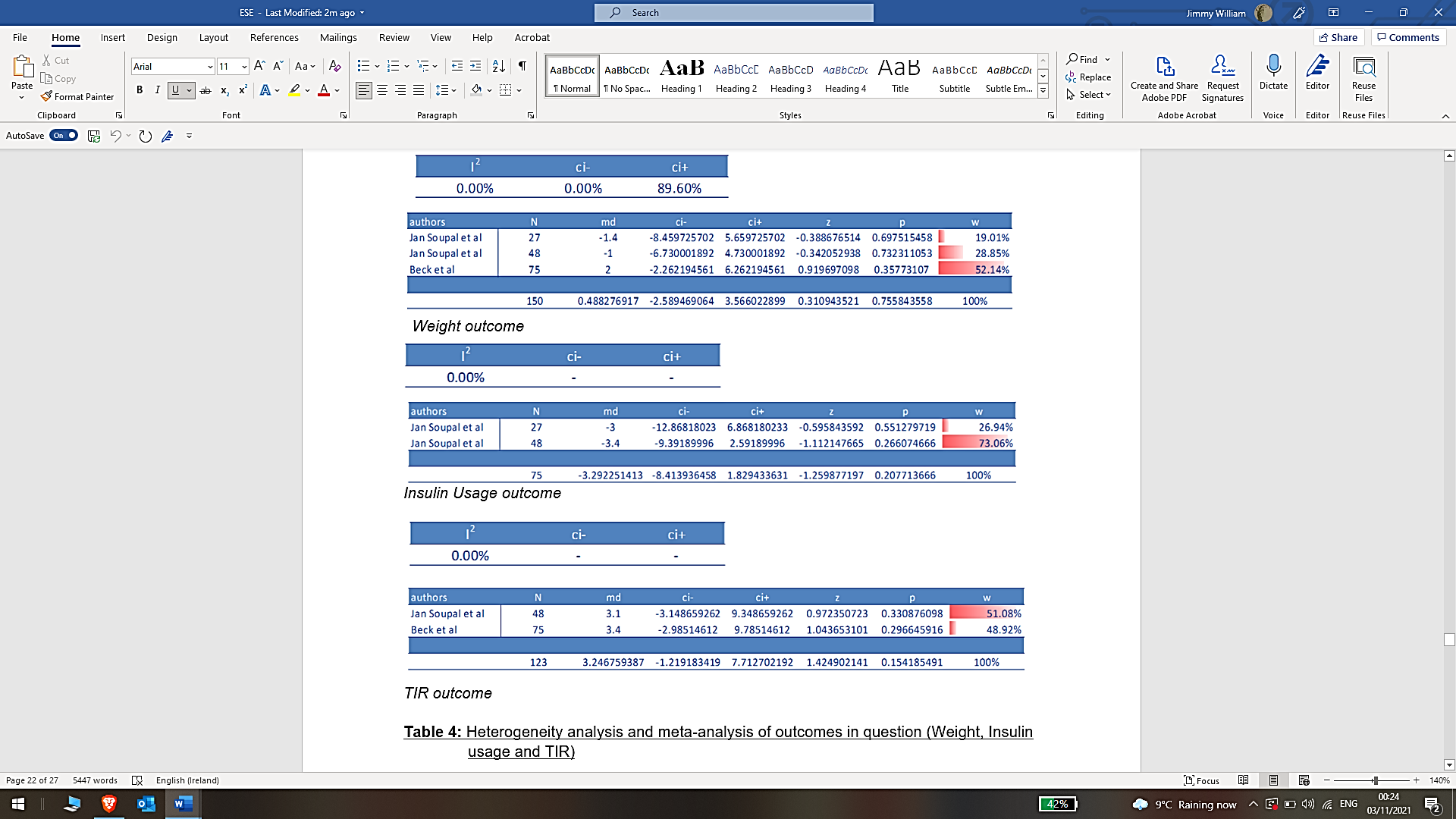


**Fig S4:** Heterogeneity analysis and meta-analysis of outcome in question
 (Weight, Insulin Usage and TIR)
